# Role of Behavioral Interventions in the Management of Obesity

**DOI:** 10.7759/cureus.18080

**Published:** 2021-09-18

**Authors:** Iyanu V Olateju, Dolly Ogwu, Matthew O Owolabi, Ugonna Azode, Felicity Osula, Richard Okeke, Ijeoma Akabalu

**Affiliations:** 1 Internal Medicine, MedStar Harbour Hospital, Baltimore, USA; 2 Psychiatry, University of Benin, Benin, NGA; 3 Pediatrics, Children's National Hospital, Washington DC, USA; 4 Clinical Research, Walden University, Minneapolis, USA; 5 Informatics, University of Wisconsin School of Medicine and Public Health, Madison, USA; 6 Physical Therapy, University of Ghana Legon, Accra, GHA; 7 Medicine and Surgery, Avalon University School of Medicine, Williemstaad, CUW; 8 Internal Medicine, The University of Chicago Medicine, Chicago, USA; 9 Internal Medicine, Ivano-Frankivsk National Medical University, Ivano-Frankivsk, UKR; 10 Medicine, Caribbean Medical University School of Medicine, Willemstad, CUW

**Keywords:** motivational interviewing, weight management, behavioral interventions for weight loss, behavioral modification, obesity

## Abstract

Overweight and obesity are the leading lifestyle-related causes of clinical and public health concerns. Health behavior change is central in obesity management. This review provides the various behavioral interventions in the management of obesity. Behavior modification is a structured method for improving lifestyle habits such as exercise, diet, and other practices that might influence behavior. This review article was based on current and older literature on behavioral approaches to target overweight/obesity. Articles about the various interventions to reduce obesity, particularly behavior interventions, were searched and explored. All the articles found to reveal behavior modification techniques, including goal-setting, self-monitoring, stimulus control, cognitive restructuring, stress management, problem-solving, and social support and information were obtained by reading the full text of the articles. The articles which did not provide any information regarding behavior interventions to reduce obesity were excluded from the review. This review discussed practical ways to incorporate behavior interventions in the management of obesity. The benefits and effectiveness of behavioral interventions on achieving and maintaining weight loss are also discussed. Patients using behavioral modification strategies to make these changes are more likely to succeed in long-term weight maintenance.

## Introduction and background

Obesity and overweight are defined as abnormal or excessive fat accumulation [1] and weight gain exceeding the standard indicator values [1,2]. Overweight and obesity are the leading lifestyle-related causes of clinical and public health concerns [2]. Obesity is often measured using the body mass index (BMI), where BMI = weight (kg)/height (m^2^) [1,3]. BMI greater than 25 is considered overweight, and over 30 is obese. Obesity is now an epidemic, with over four million people dying each year due to being overweight or obese in 2017 [1].

Bodyweight is influenced by genetic, metabolic, behavioral, environmental, cultural, and socio-economic influences [4,5]. The etiology of obesity can be observed from a clinical and public health perspective. The clinical perspective is based on individual variations (genetic and biologic variations), while the public health perspective is based on calorie intake and energy expenditure [4,5]. It is crucial to achieving a sustainable balance between energy output and input. Increasing levels of energy expenditure can be achieved by increasing physical activity levels and decreasing sources of sedentary lifestyle [4]. Therefore, overweight and obesity result mainly from a sedentary lifestyle and a negative balance between energy consumption and expenditure [4].

Obesity is associated with increased risk for metabolic disorders (such as hypertension, hyperlipidemia, dyslipidemia, type 2 diabetes), cardiovascular diseases, some types of cancer, cholelithiasis, and increased risk of morbidity and mortality [6,7]. The leading causes of death among adults with obesity include ischemic heart disease, complications from diabetes mellitus type 2, chronic obstructive pulmonary disease, and cancers [6-10]. The risk of these non-communicable diseases increases even when slightly overweight and grows more severe as the BMI climbs. There is a crucial need to develop and implement interventions that specifically achieve weight stability in those with an existing weight problem and help prevent deterioration in obesity-related comorbidities over time. Programs including nutritional, activity, and behavioral components can effectively help manage obesity/overweight [11]. Behavioral modifications targeting diet and physical activity changes are the cornerstones of interventions for weight management in overweight and obese populations [12]. Behavioral modifications are also effective in reducing weight and improving health, at least in the short term [13].

This review provides the various behavioral interventions in the management of obesity. Behavior modification is a structured method for improving lifestyle habits such as exercise, diet, and other practices that might influence behavior [14]. Behavior modification includes goal-setting, stimulus control, stress factors management, self-monitoring, cognitive restructuring, stress management, problem-solving, and support systems [14,15]. Behavioral interventions, exercise, and diet lead to more effective and sustainable weight maintenance [14,15].

## Review

Methodology

This review article was based on current and older literature on behavioral approaches to target overweight/obesity. The studies included were available publications on the topic from March 1992 through January 2021. We searched the following databases for relevant articles: Google Scholar, PubMed, ScienceDirect, and Web of Science. We identified searches using the keywords, Obesity, Weight Management, Behavioral Modification, motivational interviewing, and Behavioral Interventions for Weight Loss. In addition, we searched and explored articles cited in the primary publications for pertinent information regarding the topic. All the articles found to reveal behavior modification techniques, including goal-setting, self-monitoring, stimulus control, cognitive restructuring, stress management, problem-solving, and social support and information were obtained by reading the full text of the articles. We excluded articles that did not provide any information regarding behavior interventions to reduce obesity from the review. 

Obesity management

Efficient and successful management of obesity involves multiple treatment strategies, focusing on modifying lifestyle habits such as exercise and diet. Other methods include behavior modification, adjunctive pharmacotherapy, and surgical approach [14,16]. The recommendations for the management of obesity are represented in Table 1.

**Table 1:** Recommendations for the management of obesity

Interventions	Definition
Behavioral strategies	The use of multiple strategies like stress management, cognitive restructuring, self-monitoring, social support, and stimulus control.
Dietary intake	Reduction of caloric intake by 500 to 1,000 kcal per day; leads to a total loss of one to two pounds in body weight per week.
Physical activity	Obese patients should start with moderate-intensity physical activity like walking for 30 to 45 minutes, three to five days per week.
Adjunctive pharmacotherapy	Drug therapy should be considered in patients with BMI greater than or equal to 30 or a BMI greater than 27 with comorbidities.

Behavioral interventions in obesity management

Behavior modification is a structured method for improving lifestyle habits such as exercise, diet, and other practices that might influence obesity [14]. Most behavior modification strategies focus on: increasing awareness around triggers for problem behaviors, identifying feelings and beliefs around weight issues, increasing structure around common weight-related behaviors, providing support that enables change, and setting realistic goals for changes [11,17]. Effective behavioral strategies for weight loss is heavy on behavior change techniques such as self-monitoring, physical activity, goal setting, problem-solving, support system, stressor and stimulus control, cognitive restructuring, alternative behaviors, continuous patient-centered care, weight control, and maintenance plan, structured meal plans, meal replacements, understanding portion control, and contingency management-making specific plans for "slip-ups" and alternative behaviors [11,15,18].

Self-monitoring

Monitoring of energy consumption and expenditure and intake is the central dogma of the cognitive management of obesity. Effective self-monitoring has a direct positive relationship with weight loss [19]. Intentional surveillance and record of food and daily physical activities positively influence self-consciousness and personal behaviors. In addition, self-monitoring allows more time to self-reflect before making decisions, promoting healthier food choices [11,20]. Tools involved in self-monitoring are food diaries used to record a breakdown of daily caloric food content, nutrient groups, physical activity logs (type and duration), body weight, and mass [14]. Although patients are not always accurate in documenting their diet and exercise behaviors [19], the purpose of self-monitoring is to create awareness. Self-awareness helps patients understand how their daily choices can be beneficial or detrimental to their weight management efforts. Self-monitoring records can also provide information to identify activity contingencies targeted for intervention [21].

Goal-setting

Setting reasonable and achievable goals promotes long-term success. Goal setting effectively focuses participants' attention on behavior change and helps set a specific dietary intake and weight management goal. In selected patients (depending on their capabilities and general health), low-intensity physical activity or exercise should be started and increased gradually to moderate-intensity with a goal of 150-200 minutes per week [19,22]. Exercise compliance can be improved by promoting lifestyle activities (such as walking, hiking, riding bicycles rather than driving, taking the stairs rather than elevators), stretching, home-based endurance, and strength exercises, especially for individuals with hectic schedules [3,22].

Problem-solving

Specific problem-solving tactics help patients traverse their health-related habits. Patients are strongly advised to weigh their options before making an educated decision regarding their health [11,23]. Patients are encouraged to create and apply a personalized plan for the desired goal and measure their success against a pre-produced measurement scale. Beyond this, patients are taught to analyze their problems circumspectly and create problem-solving tactics [11]. In addition, group visits are encouraged because they promote collaborative problem-solving tactics. Individuals learn from each other’s experiences and can adopt solutions from their peers [11,23]. Problem-solving is also necessary for specific eating situations like social and emotional eating [11,23]. Social eating is typical when eating outside of the home and in restaurants. Eating in restaurants has a high chance of increasing calorie intake; patients must learn adaptative strategies for healthy eating outside of the home [11,23]. Dining out is arguably increasing but should not be abused by overeating. Understanding the negative impact of overeating might help patients control social eating. Some struggles associated with eating out are food portioning, high-calorie food, food preparation methods, and a strong desire to finish all the food on the plate [11,23]. On the other hand, emotional eating is when people eat in response to their feelings or emotions. These emotions include sadness, happiness, anger, joy, boredom, and stress. These eating responses can hurt weight maintenance [11,23].

Social support

Good social support is associated with a more sustainable weight loss [14,24]. Social supports can come from family members, community-based programs, and social activities like conferences, courses, and clubs. The community-based programs can be either weight-loss oriented or not; the essential component is a sound support system. Support systems can also be instrumental in assisting individuals to develop healthy relationships and formulate a reasonable work-life balance [14,24].

Stress management

Educating patients on various managing methods is crucial, as stress can influence eating habits [24]. Examples of stress reduction techniques are breathing exercises, muscle relaxation, mediation, and yoga. These techniques reduce tension by inhibiting stimulation of the sympathetic nervous system, thereby distracting from stressful events. Patients can also be advised to adopt other strategies, including exercise, swimming, relaxation techniques, yoga, family support, and other behavioral interventions [11,25].

Stimulus control

Stimulus control is simply conditioning, classical, and operant conditioning. It involves the identification of environmental cues associated with eating habits and inactivity. Controlling these cues can help sustain weight loss and prevent relapse [3,14,25]. Patients can implement these strategies by eating only at the dining without distractions from electronic devices, avoid storing snacks at home, bringing out active wears the night before a workout day, and a reminder note on the refrigerator or any other visible surface [3,14,25]. A collaborative patient-physician effort should develop a practical and sustainable customized stimulus control plan [14]. The primary focus should be to create a suitable environment that supports weight management goals and strategies [3,14]. The environment can be modified to encourage a healthy lifestyle by building practical and goal-oriented networks suitable for walking (such as pedestrian pathways), cycling (for bicycles), and safe recreational spaces (such as local parks and centers) [3,14,26]. It is recommended that patients remove inactivity triggers to avoid relapse [3].

Alternative behaviors

It includes recognizing triggers for overeating like a strong urge or desire to eat and identify healthy behavioral alternatives [11]. In collaboration with primary healthcare providers, patients learn to identify triggers of unhealthy behaviors and develop strategies to eliminate such behaviors. Patients are taught to gradually substitute unhealthy behaviors with healthy behaviors [11].

Cognitive restructuring

Cognitive restructuring promotes self-awareness which helps patients actively change the internal dialogue that undermines their weight loss efforts [14,16]. Cognitive restructuring is essential because many obese patients think poorly of themselves and have low expectations or are entirely ignorant of weight loss possibilities and potential benefits [14]. Cognitive restructuring helps patients to change their perspective of weight loss expectations [16,19]. The first contact with patients is usually the primary care providers; this opens room for discussing and formulating realistic and sustainable weight loss plans. This is the perfect avenue for developing a trusting physician-patient relationship and encouraging patients on their weight loss journey [11,16].

Contingency management making specific plans for slip-ups

A slip is an error. For example, Jane Doe intended to eat a wrap of chocolate but ended up eating five wraps. If Jane Doe sees this as a mistake (a slip), understands how she slipped, and treats it as a learning experience rather than dwelling on it, Jane Doe will react better (and wiser) when she is next faced with a similar temptation. The concept is to develop contingency plans to avoid repeating an error or mistake [11,23]. In the real sense, five wraps of chocolates are not necessarily going to cause obesity. However, if a slip/mistake turns into a sequence of slips, there is a high chance of relapse. Again, the belief and understanding of one’s actions influence the reaction. It is crucial to know and understand the difference between an educational experience and a failure [11,23]. A negative attitude to failed experiences impedes success, whereas a learning experience is knowledge gained [11,23]. For instance, time and seasons change, unexpected or unplanned events or incidents happen. All of these are common reasons that call for a change in an exercise routine. Rather than denying that this could ever happen, it is more prudent to stay focused on the reasons behind these problems and formulate contingency plans [11,23].

Meal planning with the help of a dietitian

For successful meal planning, it is important to assess patients' understanding and preferences. This assessment is professionally done by a registered dietitian nutritionist (RDN). RDNs work with patients to develop a practical plan that aligns with their finances, culture, and preferences while still working towards weight management and sustenance [11].

Develop specific relapse prevention techniques

Necessary components of relapse prevention include: (1) recognize social triggers that could stimulate unhealthy eating behaviors and formulate practical plans to avoid or minimize such triggers. It is also vital to generate strategies that can help prevent relapse during social events or travels [11,23]. (2) A significant cause of relapse is stress. Stress management strategies should be set up. Patients should be encouraged to discover activities that help reduce stress, such as relaxation exercises, swimming, hiking, watching movies [11,23]. (3) Staying motivated can be a hurdle; hence, it is necessary to acknowledge each milestone, success, failure, and challenge with a positive mindset. In addition, plans should be constantly assessed to set new goals and reviewed if futile [11,23].

Continuous patient-centered care 

A non-judgemental approach to patient care is the bedrock of a solid physician-patient relationship. Motivational interviewing (MI) is an effective technique used to achieve patient-centered care [11]. MI is a technique used to motivate and encourage patients to make decisions that promote their wellbeing [3]. Primary care providers play a major role in MI because they are usually the first contacted by patients. This is the perfect opportunity to educate, encourage and support patients, on their weight loss journey. In most cases, primary care providers collaborate with behavioral health specialists and other specialists like endocrinologists (per patients' health needs) to achieve desired weight loss goals. A strong medical support system promotes implementation, compliance, maintenance, and sustenance of patients' goals [11,23]. MI has the following core values: (1) Express Empathy equals skillful reflective listening is fundamental to expressing empathy. Health practitioners (HP) see the world from the individual's perspective. Acceptance from HP facilitates change in the individual [3,11]. (2) Develop discrepancy equals discrepancy between present dilemma and desired goals. HPs should help patients understand the reasoning behind their desire for change and encourage them to note it down [3,11]. (3) Roll with resistance means resistance might cause push-back, fear of the unknown, hostility, accusations, denials, and rebellion against recommendations from HPs. Once resistance is identified, HPs must avoid increasing resistance and use it constructively [3,11]. (4) Support self-efficacy means self-belief and acceptance. A self-belief is a crucial tool often maximized by HPs to effect a desired change or goal and promote change talk [3,11]. The MI strategies "OARS," aimed at promoting change talk are (1) Open-Ended Questions - helps facilitate dialogue, involve the patient in collaborative care, and give room for analytical and critical thinking. Open-ended questions are a good way to validate patients' concerns and opens the door to a potential trusting physician-patient relationship [3,27]. (2) Affirmations are statements made by HPs to genuinely motivate patients, acknowledging their strengths and achievements, replacing negativity with positivity, and reframing their failures to strengths. Affirmations encourage patients to stay persistent and determined [3,27]. (3) Reflective Listening - patiently listening to patients to understand and interpret their emotions, express empathy, and help navigate the pathway to success [3,27]. (4) Summaries - reiterate the interest of HPs in patient care, help patients identify problems while proffering workable and lasting solutions [3,27]. In a study conducted by Kelley et al. [11], MI helped reduce body mass in obese/overweight participants compared to the control group.

Effectiveness of behavioral interventions

The incorporation of behavioral modification in the management of obesity has proven to produce body mass and weight loss [3,11,16,19,26]. Over the past 20 years, multiple studies reported that behavioral interventions lasting over four months lead to an average weight loss of 0.45 kg (1 lb) per week [14,16,28,29]. The administration of multiple interventions produces a more significant weight loss [14]. After nine to ten months of behavioral treatment, a greater percentage of patients (about two-thirds) achieve and maintain weight loss [14]. In addition to exercise and diet, multiple studies consistently prove that extended behavioral treatment achieves a significant weight loss [11,14,15,24,26,28,29].

## Conclusions

Health care providers must become more involved in preventing obesity and incorporate multiple behavioral interventions in managing and preventing obesity. There is a need to transition from prescribing only diet and exercise as lifestyle interventions to a more holistic approach that includes behavioral treatment. This multidisciplinary approach to obesity management will foster patient compliance, adherence, and maintenance of lifestyle modification.

## References

[1] (2021). Obesity and overweight. https://www.who.int/news-room/fact-sheets/detail/obesity-and-overweight.

[2] Gizaw Z (2017). Prevention and control strategies of overweight and obesity. Adv Obes Weight Manage Control.

[3] (2021). Management of obesity. https://www.physio-pedia.com/Management_of_Obesity.

[4] Sharma M (2007). Behavioural interventions for preventing and treating obesity in adults. Obes Rev.

[5] Schmitz MK, Jeffery RW (2000). Public health interventions for the prevention and treatment of obesity. Med Clin North Am.

[6] LeBlanc ES, Patnode CD, Webber EM, Redmond N, Rushkin M, O'Connor EA (2018). Behavioral and pharmacotherapy weight loss interventions to prevent obesity-related morbidity and mortality in adults: updated evidence report and systematic review for the US Preventive Services Task Force. JAMA.

[7] Bogers RP, Bemelmans WJ, Hoogenveen RT (2007). Association of overweight with increased risk of coronary heart disease partly independent of blood pressure and cholesterol levels: a meta-analysis of 21 cohort studies including more than 300 000 persons. Arch Intern Med.

[8] Afshin A, Forouzanfar MH, Reitsma MB (2017). Health effects of overweight and obesity in 195 countries over 25 years. N Engl J Med.

[9] Whitlock G, Lewington S, Sherliker P (2009). Body-mass index and cause-specific mortality in 900 000 adults: collaborative analyses of 57 prospective studies. Lancet.

[10] Borrell LN, Samuel L (2014). Body mass index categories and mortality risk in US adults: the effect of overweight and obesity on advancing death. Am J Public Health.

[11] Kelley CP, Sbrocco G, Sbrocco T (2016). Behavioral modification for the management of obesity. Prim Care.

[12] Jensen MD, Ryan DH, Apovian CM (2014). 2013 AHA/ACC/TOS guideline for the management of overweight and obesity in adults: a report of the American College of Cardiology/American Heart Association Task Force on Practice Guidelines and The Obesity Society. Circulation.

[13] Apovian CM, Aronne LJ (2015). The 2013 American Heart Association/American College of Cardiology/The Obesity Society Guideline for the management of overweight and obesity in adults: what is new about diet, drugs, and surgery for obesity?. Circulation.

[14] Poston WS, Foreyt JP (2000). Successful management of the obese patient. Am Fam Physician.

[15] (2021). Behavioural interventions for management of overweight and obesity in adults. https://www.obesityevidencehub.org.au/collections/treatment/behavioural-interventions-for-the-management-of-overweight-and-obesity-in-adults.

[16] Teixeira PJ, Marques MM (2017). Health behavior change for obesity management. Obes Facts.

[17] Stunkard AJ (1996). Current views on obesity. Am J Med.

[18] (2021). Adult weight management (AWM) guideline (2014). https://www.andeal.org/topic.cfm.

[19] Dalle Grave R, Calugi S, Centis E, El Ghoch M, Marchesini G (2011). Cognitive-behavioral strategies to increase the adherence to exercise in the management of obesity. J Obes.

[20] Baker RC, Kirschenbaum DS (1993). Self-monitoring may be necessary for successful weight control. Behavior Therapy.

[21] Lichtman SW, Pisarska K, Berman ER (1992). Discrepancy between self-reported and actual caloric intake and exercise in obese subjects. N Engl J Med.

[22] Fabricatore AN (2007). Behavior therapy and cognitive-behavioral therapy of obesity: is there a difference?. J Am Diet Assoc.

[23] Veilleux JC, Skinner KD (2015). Smoking, food, and alcohol cues on subsequent behavior: a qualitative systematic review. Clin Psychol Rev.

[24] Kayman S, Bruvold W, Stern JS (1990). Maintenance and relapse after weight loss in women: behavioral aspects. Am J Clin Nutr.

[25] Vine M, Hargreaves MB, Briefel RR, Orfield C (2013). Expanding the role of primary care in the prevention and treatment of childhood obesity: a review of clinic- and community-based recommendations and interventions. J Obes.

[26] Foreyt JP, Goodrick GK (1993). Evidence for success of behavior modification in weight loss and control. Ann Intern Med.

[27] (2021). Motivational interviewing: open questions, affirmation, reflective listening, and summary reflections (OARS) | The Homeless Hub. https://www.homelesshub.ca/resource/motivational-interviewing-open-questions-affirmation-reflective-listening-and-summary.

[28] Jacob JJ, Isaac R (2012). Behavioral therapy for management of obesity. Indian J Endocrinol Metab.

[29] Butryn ML, Webb V, Wadden TA (2011). Behavioral treatment of obesity. Psychiatr Clin North Am.

